# Immunity after COVID-19 and vaccination: follow-up study over 1 year among medical personnel

**DOI:** 10.1007/s15010-021-01703-9

**Published:** 2021-09-25

**Authors:** Vivian Glück, Sonja Grobecker, Josef Köstler, Leonid Tydykov, Manuela Bertok, Tanja Weidlich, Christine Gottwald, Bernd Salzberger, Ralf Wagner, Florian Zeman, Michael Koller, André Gessner, Barbara Schmidt, Thomas Glück, David Peterhoff

**Affiliations:** 1 Institute for Clinical Microbiology and Hygiene, University Hospital Regensburg, Regensburg, Germany; 2 Kliniken Südostbayern AG, Klinikum Traunstein, Traunstein, Germany; 3 Department for Infection Control and Infectious Diseases, University Hospital Regensburg, Regensburg, Germany; 4 Institute for Medical Microbiology and Hygiene, University of Regensburg, Regensburg, Germany; 5 Center for Clinical Studies, University Hospital Regensburg, Regensburg, Germany

**Keywords:** COVID-19, SARS-CoV-2, Cellular immunity, Antibody-mediated immunity, SARS-CoV-2-vaccination

## Abstract

**Background:**

The long-term course of immunity among individuals with a history of COVID-19, in particular among those who received a booster vaccination, has not been well defined so far.

**Methods:**

SARS-CoV-2-specific antibody levels were measured by ELISA over 1 year among 136 health care workers infected during the first COVID-19 wave and in a subgroup after booster vaccination approximately 1 year later. Furthermore, spike-protein-reactive memory T cells were quantified approximately 7 months after the infection and after booster vaccination. Thirty healthy individuals without history of COVID-19 who were routinely vaccinated served as controls.

**Results:**

Levels of SARS-CoV-2-specific IgM- and IgA-antibodies showed a rapid decay over time, whereas IgG-antibody levels decreased more slowly. Among individuals with history of COVID-19, booster vaccination induced very high IgG- and to a lesser degree IgA-antibodies. Antibody levels were significantly higher after booster vaccination than after recovery from COVID-19. After vaccination with a two-dose schedule, healthy control subjects developed similar antibody levels as compared to individuals with history of COVID-19 and booster vaccination. SARS-CoV-2-specific memory T cell counts did not correlate with antibody levels. None of the study participants suffered from a reinfection.

**Conclusions:**

Booster vaccination induces high antibody levels in individuals with a history of COVID-19 that exceeds by far levels observed after recovery. SARS-CoV-2-specific antibody levels of similar magnitude were achieved in healthy, COVID-19-naïve individuals after routine two-dose vaccination.

**Supplementary Information:**

The online version contains supplementary material available at 10.1007/s15010-021-01703-9.

## Introduction

Comparable to other infectious viral diseases, at least temporary immunity is assumed for most individuals after recovery from a SARS-CoV-2 infection [1, 2]. However, investigations into the time course of SARS-CoV-2-directed immunity after infection have yielded conflicting results so far. It has become clear that the strength of the immune response underlies considerable interindividual variability, and decreases with age and over time [3–11]. Reinfections have rarely been reported, and generally follow mild courses. However, surrogate parameters for and the duration of protection from reinfection have not been defined so far. Only few studies addressed time periods longer than 6–8 months and especially parameters of cellular immunity have been insufficiently studied to date [5–7, 9–11].

Similar to other countries, health care workers in Germany are overrepresented among COVID-19-infected individuals [12]. Here, we describe the course of antibody-mediated and cellular SARS-CoV-2-directed immunity over a time period of 1 year in a cohort of hospital employees, who were infected with SARS-CoV-2 during the first COVID-19 wave in spring 2020 and recovered subsequently. Furthermore, we describe the effects of booster vaccination on SARS-CoV-2-directed antibodies and memory T cells in a subgroup of the cohort in comparison to healthy, COVID-19-naïve controls, who received vaccination against COVID-19 with a standard two-dose schedule.

## Methods

### Study cohort and blood sampling

Employees of the Kliniken Südostbayern Hospital Network (Bavaria, Germany) who recovered from a RT-PCR-confirmed COVID-19 episode between April and June 2020 were asked to participate in the prospective cohort study. After written informed consent, directly after recovery, and after approximately 12, 30 and 48 weeks, participants were asked to provide a serum sample (S-Monovette, Sarstedt, Nümbrecht, Germany), and additionally at approximately 30 weeks also a heparin-anticoagulated whole-blood sample for analysis of cellular immunity (LH Monovette, Sarstedt, Nümbrecht, Germany). At each blood sampling date, participants were asked to report their COVID-19-specific symptoms in structured questionnaires.

Approximately 1 year after COVID-19, when vaccines had been approved by heath officials and become available, participants were offered a one dose booster vaccination against COVID-19, according to the recommendations of the German vaccination advisory board (STIKO) [13]. Those who agreed to be booster-vaccinated were asked to provide another serum and whole blood sample at least 14 days thereafter.

Healthy employees without evidence of prior COVID-19 according to symptoms, negative anti-SARS-CoV-2 antibodies and repeated consistently negative SARS-CoV-2 PCR-tests served as controls and underwent the standard two-dose vaccine schedule between February and April 2021 in accordance with STIKO recommendations. They were asked to provide a serum sample immediately prior to the second vaccination and a serum and a whole-blood sample at least 14 days thereafter.

Serum was obtained by centrifugation within 6 h after drawing the blood sample and stored at − 20 °C until analysis. Mononuclear cells (PBMC) were isolated by density gradient centrifugation (Leucosep^®^-vials, Greiner Bio-One, Frickenhausen, Germany) und with lymphocyte-separation media (Anprotec, Bruckberg, Germany).

The study was approved by the University of Regensburg ethic committee (reference number 20-1896-101).

### Detection of SARS-CoV-2-spike-protein’s receptor-binding domain-specific antibodies by ELISA

Anti-SARS-CoV-2-specific antibody levels in serum were detected by an ELISA utilizing the SARS-CoV-2-spike-protein’s receptor-binding domain (RBD) as antigen, as previously described [14]. The assay is able to detect IgM-, IgA- and IgG-SARS-CoV-2 antibody responses separately with high specificity and sensitivity and the detected antibody levels were shown to correlate well with the virus neutralization capacity of the respective serum sample. ELISA results were expressed as optical densities of the sample/background ratios (signal/cutoff; S/CO), and ELISA readings ≤ 1.0 S/CO were considered negative. 1.0 < S/CO ≤ 3.0, 3.0 < S/CO ≤ 6.0, and S/CO > 6.0 were considered low, intermediate and high antibody titers, respectively. S/CO differences > 0.1 were defined as antibody titer increase or decrease of the antibody level. The high antibody levels in serum samples after vaccination, respectively, booster vaccination were titrated in eight steps of twofold dilutions, starting at a dilution of 1:200.

### Detection of SARS-CoV-2-specific spike-protein-reactive memory T cells by ELISpot

MultiScreen-IP-plates (0.45 μm, Merck Chemicals, Darmstadt, Germany) were activated with 35% ethanol and coated with 10 µg/ml anti-human IFN-γ (1-D1K, Mabtech, Nacka Strand, Sweden). Prior to addition of PBMCs, the plates were blocked with RPMI-medium and 10% FKS for 1 h at 37 °C, 5% CO_2_ in a humidified incubator. Stimulation of the cells was done with 1 µg/ml PepTivator SARS-CoV-2 Prot S (Miltenyi Biotec B.V. & Co. KG, Bergisch Gladbach, Germany). Stimulation with 6.7 µg/ml phytohemagglutinin (PHA-L, PAN Biotech, Aidenbach, Germany) served as positive control. After 16–18-h incubation, 1 µg/ml biotinylated anti-human INF-γ monoclonal antibody (7-B6-1, Mabtech, Nacka Strand, Sweden) was added and incubated for another 2 h at room temperature. This was followed by addition of streptavidin–alkaline-phosphatase-conjugate (Mabtech, Nacka Strand, Sweden) 1:1000 in DPBS and incubation for another hour. Finally, the plates were covered with substrate solution (BCIP/NBTplus, Oxford Immunotec, Abingdon, UK) for 7 min. Analysis of the dried plates was done with an ELISpot Reader System (iSpot Robot and ELISpot Reader 7.0, AID, Straßberg, Germany).

### Statistics

Analysis of the data was explorative. Descriptive statistics were calculated from raw data using SPSS (SPSS Statistics 26, IBM, New York/USA) und GraphPad Prism (GraphPad Prism for Windows 9.0; GraphPad, San Diego/USA). Mann–Whitney *U* test was used for nonparametric comparison of two groups and Kruskal–Wallis test with Dunn’s correction for multiple testing for comparison of multiple groups, as indicated. Comparison of paired samples was done with the Wilcoxon signed-rank test. Graphs were generated with Graphpad Prism.

## Results

### Clinical course

Between April 9, 2020 and June 03, 2020, 136 employees of the hospital network were enrolled in the cohort. These were majorly directly involved in patient care, of younger/middle age (median 38 (inter-quartile range, IQR 29–49) years, 64% female) and generally healthy (15/130 (12%) with a chronic condition according to self-assessment). None of the study participants required inpatient care during the acute COVID-19-illness. 10/130 (8%), 50/130 (38%) and 70/130 (54%) of study participants rated their symptoms during the acute COVID-19 illness as severe, moderate and minor, respectively. Detailed characteristics of the study cohort and the correlation between symptoms, demographic data and antibody levels over the first 8 months have already been reported in detail [3]. During the whole observation period, none of the study participants developed a symptomatic reinfection. The sampling protocol and the number of participants at each time point of blood sampling are shown in Fig. S1.

### Course of antibody levels

**Fig. 1:**
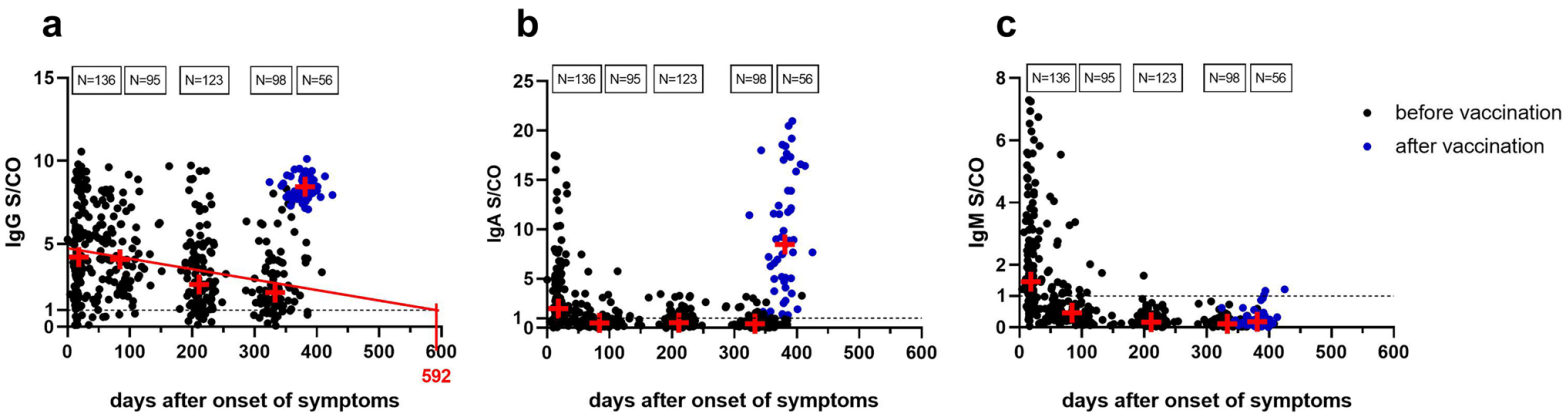
Course of anti-SARS-CoV-2-S protein-directed IgG (**a**), IgA (**b**) and IgM (**c**) antibody titers over 1 year after onset of symptoms (black closed circle), as well as after the booster vaccination (blue closed circle). Median values (red plus) and linear regression line of the IgG course (solid red line) are shown

The course of SARS-CoV-2-specific antibody levels over 1 year after COVID-19 symptom onset showed a decrease with wide interindividual variation. Only 7/136 (5%) of study participants developed no measurable antibody response directly after infection. Nearly 1 year after symptom onset (median 333 (IQR 320–345) days) the proportion of seronegative individuals increased to 21/98 (21%). Extrapolation of the decay of average IgG-anti-SARS-CoV-2 antibody levels allows the estimate that, assuming further linear decay of the antibody levels, nearly 600 days after the onset of symptoms half of the individuals in this study will have antibody levels below the cutoff of the ELISA assay (Fig. 1a).

Anti-SARS-CoV-2-IgA-antibody levels declined more rapidly than IgG-antibodies and were nearly 1 year after symptom onset above the detection limit in only in 19/98 (19%) of study participants (Fig. 1b). IgM-antibody levels were short lived and detectable only briefly after infection (Fig. 1c).

### Memory T cell counts

SARS-CoV-2-spike-protein peptide reactive, interferon γ-producing mononuclear cells (mainly memory T cells [15]) were detected by ELISpot at a median of 211 (IQR 202–221) days after COVID-19 infection in 66% of study participants. Counts of these cells did not correlate with the antibody levels specific for the same antigen and measured at the same point in time (Spearman’s rho = 0.18). At this date, approximately 7 months after the infection, only 8/118 (7%) of study participants did not show any measurable antibody-mediated or cellular immunity against SARS-CoV-2.

### Antibody-mediated and cellular immunity after vaccination

Between December 31, 2020 and April 29, 2021, 56/136 (41%) of study participants received a booster vaccination, 19 (34%), 17 (30%) and 20 (36%) the vaccines Vaxzevria (Oxford/Astra-Zeneca), Comirnaty (BioNTech) and Spikevax (Moderna), respectively. The 30 control subjects did not differ from the study cohort (median age 41 (IQR 27–50) years, 67% female) and all except two were vaccinated with the Comirnaty vaccine.

**Fig. 2:**
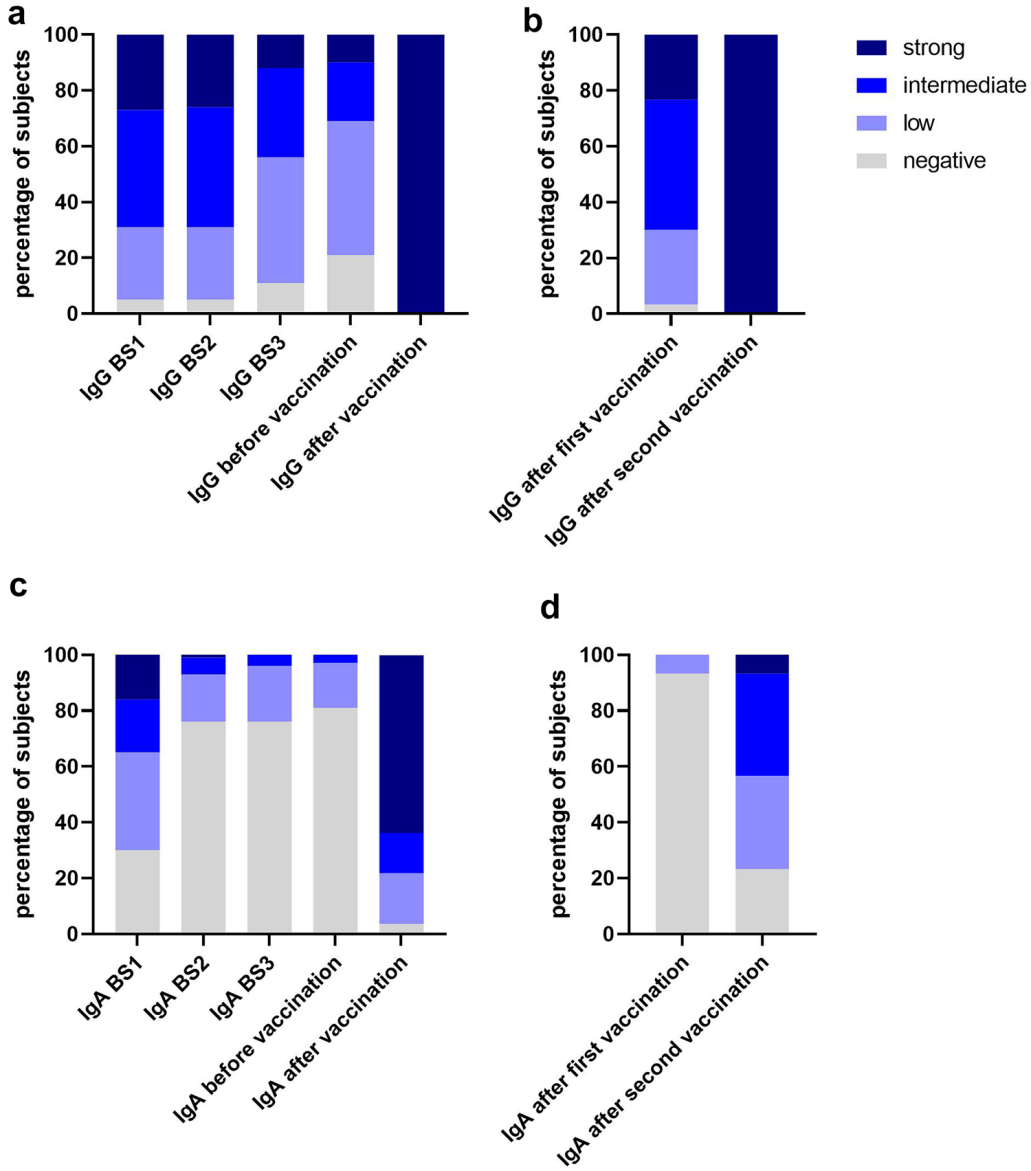
Percentage of participants in study group (**a**, **c**) or control group (**b**, **d**) with strong, intermediate, low or negative IgG- (**a**, **b**) or IgA-antibody titers (**c**, **d**) over 1 year after onset of symptoms and after booster vaccination (BS1, first blood sample after infection; BS2, blood sample after 12 weeks; BS3, blood sample after 30 weeks; before vaccination, blood sample after 48 weeks). Classification of anti-SARS-CoV-2 antibody titers: strong, > 6 S/CO; intermediate, 3 < *x* ≤ 6 S/CO; low, 1 < *x* ≤ 3 S/CO; negative, ≤ 1 S/CO

The booster vaccination induced among the study participants with previous COVID-19 a very strong increase in anti-SARS-CoV-2-IgG-antibody levels. Determined a median of 42 (IQR 22–59) days after receipt of the booster dose, antibody levels reached significantly higher levels as immediately after infection (Fig. 1a, *p* < 0.001; Fig. 2a).

Similarly, after booster vaccination 54/56 (96%) of the study participants developed anti-SARS-CoV-2-IgA-antibody, that were significantly higher than directly after the infection (Fig. 1b, *p* < 0.001). In particular, 36/56 (64%) showed high IgA-levels (Fig. 2c), the COVID-19-naïve controls, however, showed weaker IgA-responses after the standard two-dose vaccination (Fig. 2d). SARS-CoV-2-specific IgM-antibodies were detected in only 3/56 (5%) of study participants after booster vaccination and in none of the vaccinated controls (Fig. 1c).

**Fig. 3:**
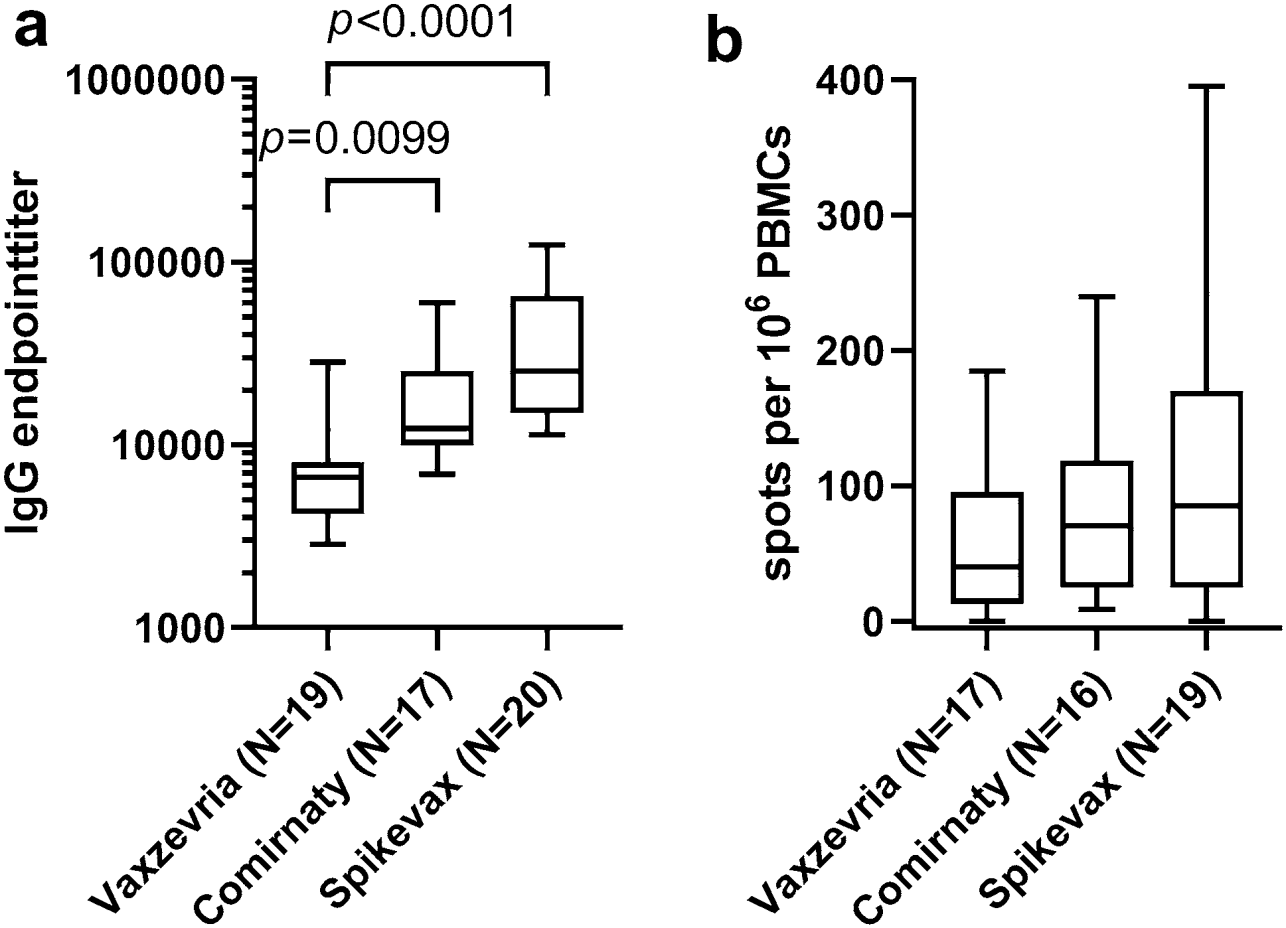
Comparison of anti-SARS-CoV-2 antibody titers (**a**) and SARS-CoV-2-S-protein-reactive memory T cell counts (**b**) in post-COVID-19 subjects after booster vaccination with Vaxzevria, Comirnaty, and Spikevax vaccines (Kruskal–Wallis test, Dunn’s correction for multiple testing)

**Fig. 4:**
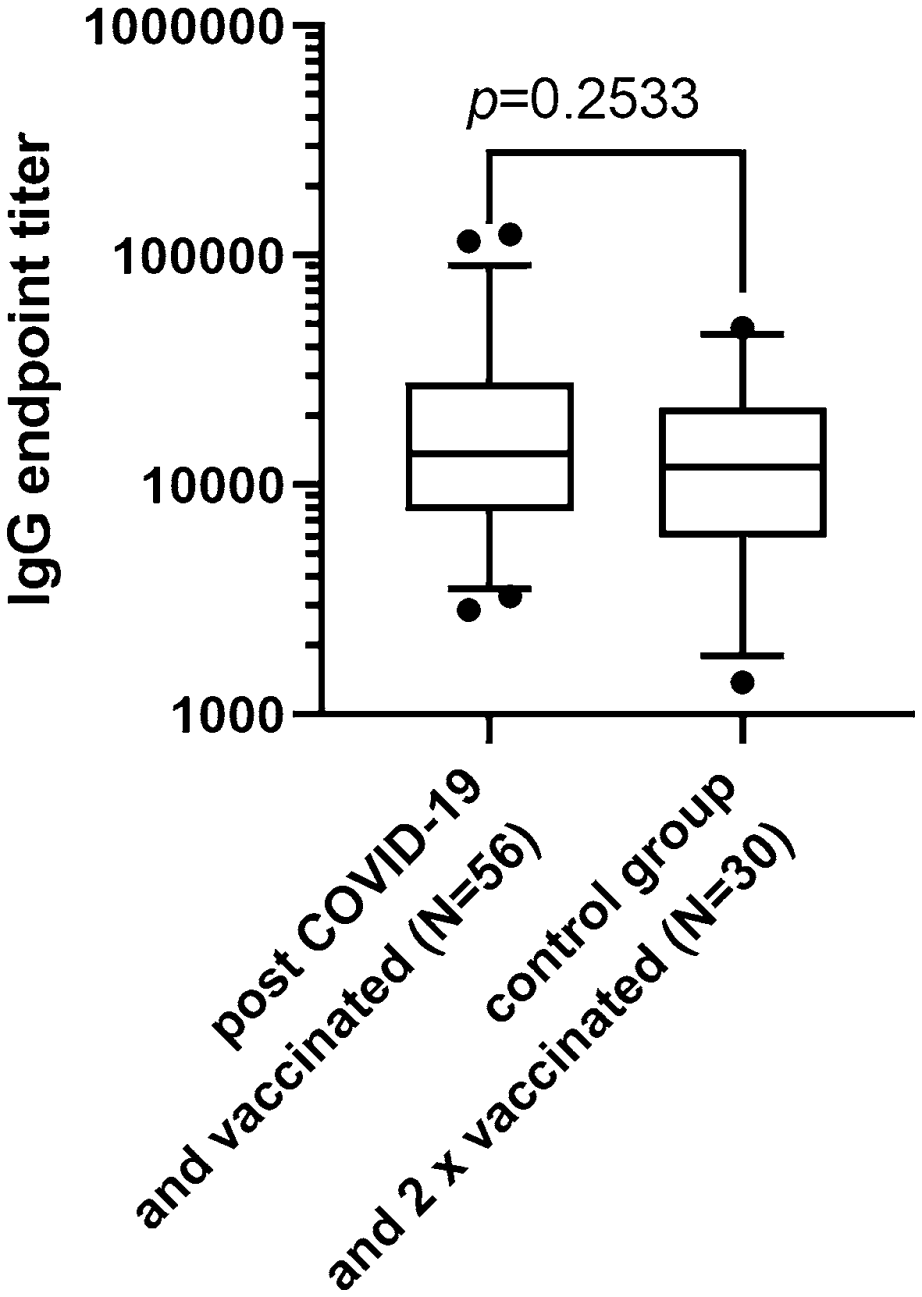
Anti-SARS-CoV-2-specific antibody titers (5/95 percentile) of post COVID-19 subjects after booster vaccination in comparison to control group subjects after dual vaccination (Mann–Whitney *U* test)

While booster-vaccinated study participants generally developed high anti-SARS-CoV-2-IgG-titers, mRNA vaccine (Comirnaty and Spikevax) recipients showed even higher antibody titers than Vaxzevria recipients (Fig. 3a; Vaxzevria vs. Comirnaty *p* = 0.0099; Vaxzevria vs. Spikevax *p* < 0.001). Antibody titers in COVID-19-naïve controls with complete vaccination did not differ significantly from study participants after booster vaccination (Figs. 1a, 4) and were also significantly above the levels of study participants immediately after COVID-19 infection (*p* < 0.001).

**Fig. 5:**
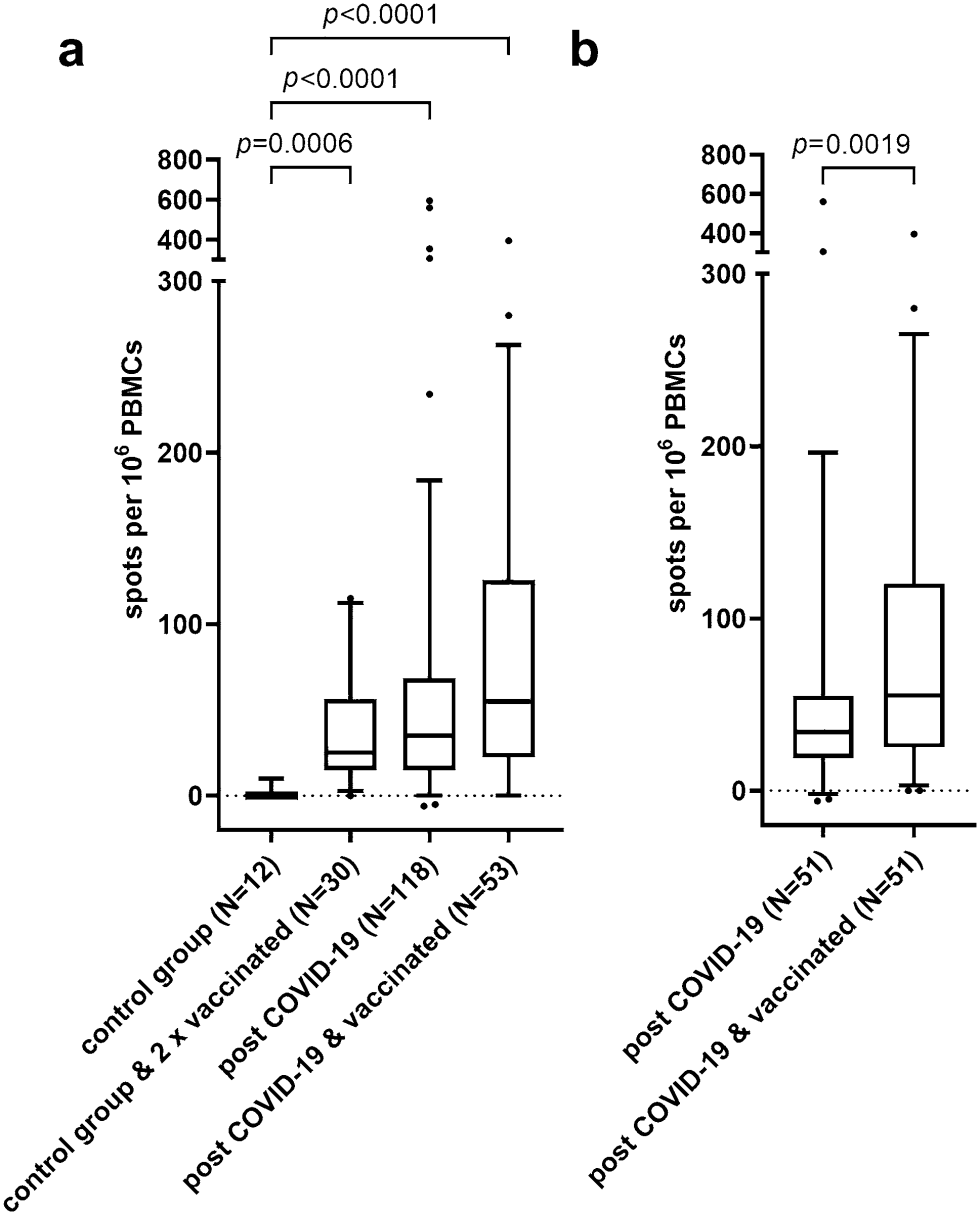
Comparison of anti-SARS-CoV-2-specific IFNy-secreting cells in SARS-CoV-2-naïve control group subjects before vaccination, after dual vaccination, in post-COVID-19 subjects approx. 7 months after onset of symptoms and in post-COVID-19 subjects after booster vaccination. **a** Comparison of all datasets (Kruskal–Wallis test, Dunn’s correction for multiple testing). **b** Comparison of paired datasets of IFNy-secreting cells in post-COVID-19 subjects 30 weeks after infection and after booster vaccination (Wilcoxon signed-rank test)

Elevated SARS-CoV-2-S-protein-reactive memory T cell counts were detectable both in individuals with previous COVID-19 and in COVID-19-naïve vaccine recipients (Fig. 5a). A booster vaccination led in study participants to a significant increase in SARS-CoV-2-S-protein-specific memory T cell counts (Fig. 5c; *p* = 0.002). SARS-CoV-2-S-protein-reactive memory T cell counts were numerically highest after vaccination with Spikevax and lowest after Vaxzevria, but the differences were not statistically significant (Fig. 3b). SARS-CoV-2-naïve controls after complete vaccination showed numerically, but not statistically lower SARS-CoV-2-S-protein-specific memory T cell counts compared to study participants after infection or after booster vaccination (Fig. 5b).

SARS-CoV-2-S-protein-specific memory T cell counts did not correlate with antibody levels after COVID-19 infection, after booster vaccination of individuals with previous COVID-19 and after complete vaccination in controls.

## Discussion

This work describes the course anti-SARS-CoV-2-directed immune responses in a cohort of generally healthy hospital employees with mostly mild COVID-19. Our results confirm previous reports indicating a continuous decay of antibody levels with time. Surprisingly, counts of SARS-CoV-2-spike-protein-reactive, interferon γ-producing mononuclear cells (mainly memory T cells), induced both through natural infection and through vaccination, did not correlate at all with antibody levels against the same antigen at the same time. It is undetermined to date whether antibody-mediated or cellular immunity is more relevant for mediating protection against SARS-CoV-2. Approximately 7 months after the infection, we could demonstrate that the majority of study participants (93%) showed measurable antibody-mediated and/or cellular immunity against SARS-CoV-2. This number compares well with published reinfection rates after natural infection or vaccination [2, 16].

Published literature estimates the anti-SARS-CoV-2-directed IgG-antibody half-life between 85 and 158 days [17–19]. However, extrapolation of the results from this cohort rather point towards an even longer half-life of approximately 300 days assuming linear decay or even longer when expecting negative exponential decay. This would mean that approximately 1½–2 years after infection, half of those who recovered from COVID-19 (and did not receive a booster vaccination) would no longer have measurable antibody-mediated immunity. This time course of immunity is in line with a recent study by Vanshylla et al. and extrapolations from observations for other coronaviruses [20, 21].

A booster vaccination, which was administered to half of the cohort not until 11–12 months after infection (because the vaccines became only then available, unlike the actual STIKO-recommendation that now advocates only 4 weeks after infection), induced a pronounced rise in SARS-CoV-2-directed IgG- and IgA-antibody levels. This indicates that natural SARS-CoV-2 infection obviously leaves a considerable number of memory B cells and plasma cells that allow for a rapid reuptake of antibody production in large amounts, even if levels have become low or even undetectable before, as has been shown recently [22–24]. In line with previous reports, we could demonstrate antibody levels of similar magnitude both in booster-vaccinated individuals with previous COVID-19 and in fully vaccinated controls [24, 25].

This reassuring finding stands in contrast to a recent publication from Italy that describes lower antibody levels in fully vaccinated COVID-19-naïve individuals [26]. Other groups showed that a second booster dose given after the first booster vaccination to patients with previous COVID-19 did not increase antibody levels further [25, 27].

In booster-vaccinated individuals with previous COVID-19, we demonstrated significantly higher antibody levels in mRNA vaccine recipients than in recipients of the vector-based vaccine Vaxzevria. SARS-CoV-2-S-protein-reactive memory T cell counts were also numerically, but not significantly higher after vaccination with the mRNA vaccines than after vaccination with Vaxzevria. This observation confirms indirect evidence from a meta-analysis of immunological data from phase I and II studies together with effectiveness data from phase III studies for the various vaccines [28] that also demonstrated weaker immune responses after vaccination with vector-based vaccines.

The profound increase observed not only in SARS-CoV-2-directed IgG- but also in IgA-antibodies after booster vaccination deserves consideration, as IgA-type antibodies are thought to play an important role in mucosal immunity and COVID-19 naïve individuals developed considerably weaker specific IgA-antibody responses after vaccination (Fig. 2c, d). Whether this is of clinical relevance for protection from reinfection remains to be demonstrated [29].

In this study T cell-mediated responses were measured by ELISpot, detecting mainly T memory cells. Memory T cells could not be demonstrated in all individuals and no correlation was observed with the antibody responses, but the vast majority of individuals in this cohort showed SARS-CoV-2-specific antibodies and/or memory T cells. The memory T cell response was numerically, but not statistically lower among vaccinated COVID-19-naïve controls than among individuals with past COVID-19, an observation that should be further studied. To date it is still unclear, whether cellular or antibody-mediated immunity is the dominant factor in long-term immunity against SARS-CoV-2, and whether the differences demonstrated between the various vaccines are of clinical relevance [18, 30, 31].

This is a prospective cohort study with voluntary participation of the individuals enrolled, not a randomized placebo-controlled trial and as such has limitations. Furthermore, we did not plan to formally evaluate vaccine effectiveness. A strength of the study is the long observation period of a cohort of generally healthy individuals with low dropout rate (see Fig. S1), covering the complete progress of the pandemic so far. This provides a “real-life” picture of the antibody level dynamics over time, and after vaccination. No reinfections were observed among the study participants, but given the generally low reinfection rates, the number of individuals enrolled is clearly insufficient for drawing conclusions which of the studied immunological parameters might indicate protection from reinfection. Such parameters are an important topic of current multicenter studies with considerably higher numbers of participants.

## Supplementary Information

Below is the link to the electronic supplementary material.

Supplementary file1 (PDF 213 KB)
