# Supplementary material for: Immunity after COVID-19 and vaccination: follow-up study over 1 year among medical personnel
Source: Infection. 2021 Sep 25;50(2):439–46. doi: 10.1007/s15010-021-01703-9 (PMC8475821; doi:10.1007/s15010-021-01703-9)
Supplement: Supplementary file 1 — Supplementary file1 (PDF 213 KB) [file 15010_2021_1703_MOESM1_ESM.pdf]

Supplementary material

Immunity after COVID-19 and vaccination: follow-up study over one year among medical personnel

Vivian Glück[1], Sonja Grobecker[1], Josef Köstler[1], Leonid Tydykov[1], Manuela Bertok[2], Tanja Weidlich[2], Christine Gottwald[2], Bernd Salzberger[3], Ralf Wagner[1,4], Florian Zeman[5], Michael Koller[5], André Gessner[1,4], Barbara Schmidt[1,4], Thomas Glück[2], David Peterhoff[1,4]*

[1] Institute for Clinical Microbiology and Hygiene, University Hospital Regensburg, Regensburg, Germany

[2] Kliniken Südostbayern AG, Klinikum Traunstein, Traunstein, Germany

[3] Department for Infection Control and Infectious Diseases, University Hospital Regensburg, Regensburg, Germany

[4] Institute for Medical Microbiology and Hygiene, University of Regensburg, Regensburg, Germany

[5] Center for Clinical Studies, University Hospital Regensburg, Regensburg, Germany

* Correspondence: david.peterhoff@ur.de

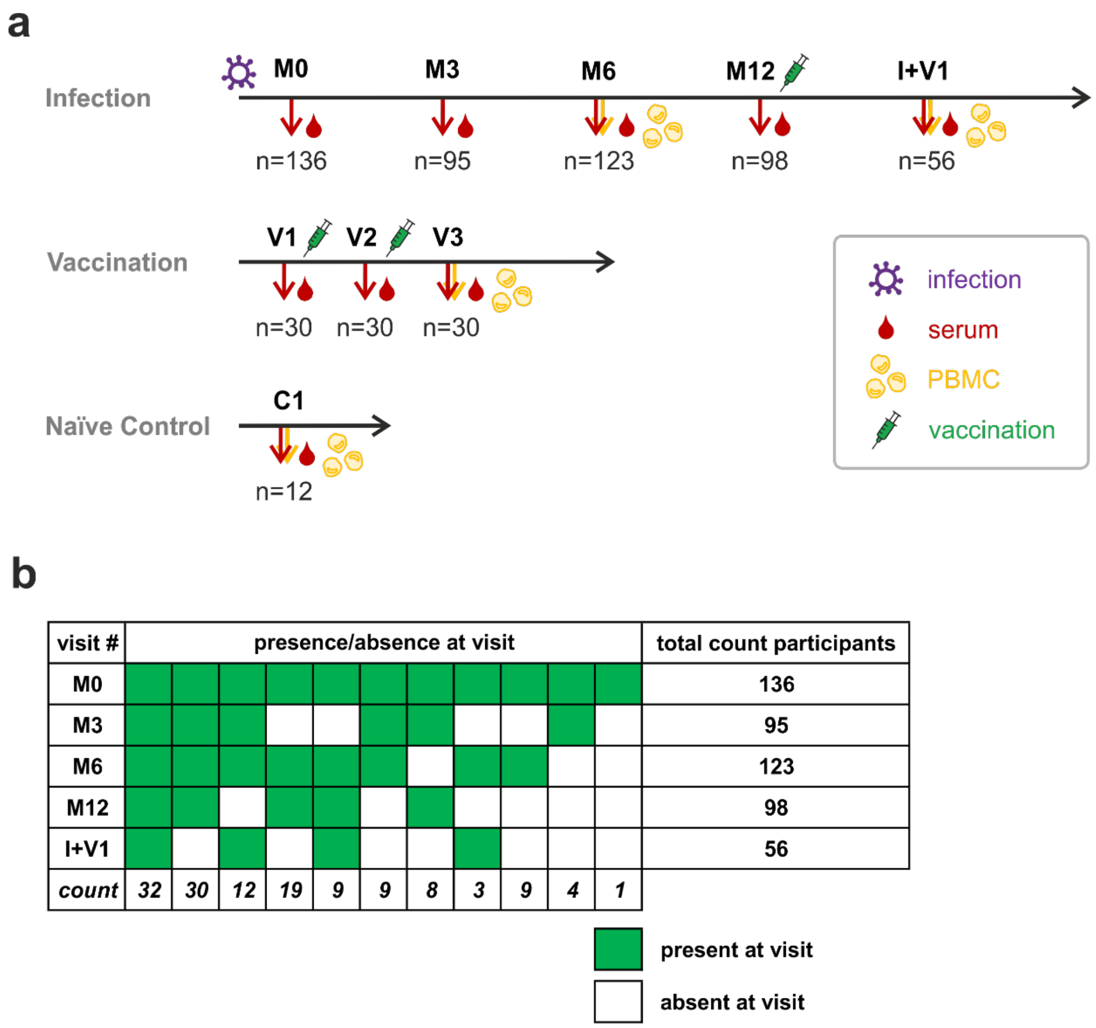

Fig. S1: Sampling protocol and number of participants at each time point. (a) Sampling timelines and sampling material for the COVID-19 convalescent group ("Infection"), control group of SARS-CoV-2 naïve vaccinees ("Vaccination") and control group of SARS-CoV-2 naïve subjects ("Naïve control"), respectively. (b) Table summarizing the counts of the combination of presences during the five study visits of the COVID-19 convalescent group. M0: sample 3 weeks (median) after onset of symptoms; M3: sample 12 weeks (median) after onset of symptoms; M6: sample 30 weeks (median) after onset of symptoms; M12: sample 48 weeks (median) after onset of symptoms; I+V1: sample 42 (median) days after vaccination; V1: sample before first vaccination; V2: sample before second vaccination; V3: sample 15 days (median) after second vaccination.
